# Berberine: An Important Emphasis on Its Anticancer Effects through Modulation of Various Cell Signaling Pathways

**DOI:** 10.3390/molecules27185889

**Published:** 2022-09-10

**Authors:** Saleh A. Almatroodi, Mohammed A. Alsahli, Arshad Husain Rahmani

**Affiliations:** Department of Medical Laboratories, College of Applied Medical Sciences, Qassim University, Buraydah 52571, Saudi Arabia

**Keywords:** berberine, cancer, cell signaling pathways, bioavailability, synergistic effects

## Abstract

Cancer is the most commonly diagnosed type of disease and a major cause of death worldwide. Despite advancement in various treatment modules, there has been little improvement in survival rates and side effects associated with this disease. Medicinal plants or their bioactive compounds have been extensively studied for their anticancer potential. Novel drugs based on natural products are urgently needed to manage cancer through attenuation of different cell signaling pathways. In this regard, berberine is a bioactive alkaloid that is found in variety of plants, and an inverse association has been revealed between its consumption and cancer. Berberine exhibits an anticancer role through scavenging free radicals, induction of apoptosis, cell cycle arrest, inhibition of angiogenesis, inflammation, PI3K/AKT/mammalian target of rapamycin (mTOR), Wnt/β-catenin, and the MAPK/ERK signaling pathway. In addition, synergistic effects of berberine with anticancer drugs or natural compounds have been proven in several cancers. This review outlines the anticancer effects and mechanisms of action of berberine in different cancers through modulation of various cell signaling pathways. Moreover, the recent developments in the drug delivery systems and synergistic effect of berberine are explained.

## 1. Introduction

Cancer is a multifactorial disease and is featured by the unrestricted proliferation of abnormal cells with the potentiality to spread to the whole body [1]. The death percentage due to this dreadful disease is rapidly growing and, by 2030, it is predicted that there will be around 26 million new cancer cases and 17 million cancer deaths per year [2]. The mechanisms behind tumor development are not clearly understood. However, a number of cancer-causing agents are known to play a significant role in tumor development and proliferation. Numerous factors can lead to the development of cancer, which can arise from endogenous factors, including genetic predisposition or age, unregulated hormones, inherited mutations, and immune conditions. The exogenous factors or acquired factors include nutrition, environment, lifestyle, diet, tobacco, obesity, exposure to sun, chemicals, and radiation, in addition to infectious organisms, or combinations of these [3,4,5,6].

Alteration in the expression pattern of tumor suppressor genes, apoptotic genes, and oncogenes shows a vital role in the patho-physiological mechanisms of tumors [7,8]. The currently used chemotherapeutic drugs, including cisplatin, 5-fluorouracil, and doxorubicin, can be effective but also cause severe adverse effects, damage the normal cells and altering the expression pattern of various cell signaling pathway genes. Effective natural anticancer drugs or drugs based on medicinal plants with lower toxicity and side effects are urgently needed. As per the previous plentiful experimental data, it has been reported that phytochemicals such as flavones, polyphenols, and flavonoids possess significant anticancer properties [9].

**Figure 1:**
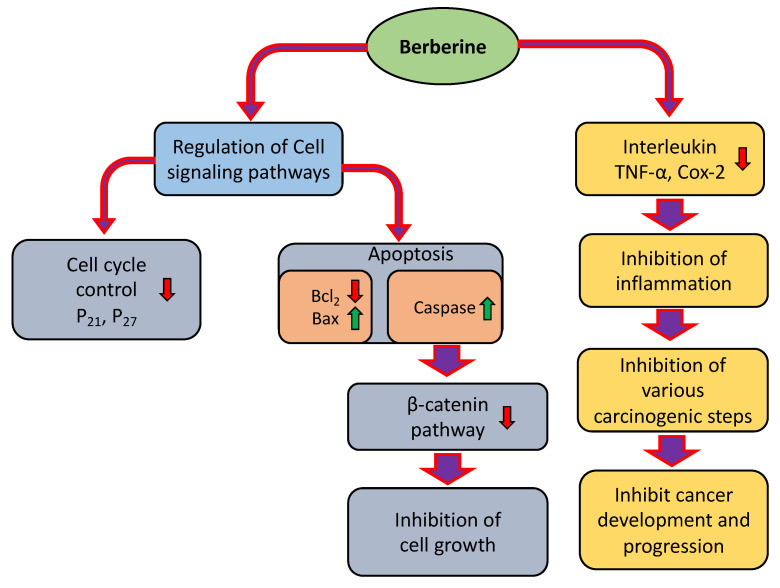
Anticancer effects of berberine through modulation of various cell signaling pathways.

Berberine (BRB) is an isoquinoline alkaloid commonly extracted from a medicinal herb, *Rhizoma coptidis*, and plays a significant role in the inhibition of pathogenesis via suppression of intracellular reactive oxygen species (ROS) generation. This alkaloid stimulates the pro-inflammatory responses, through constraining various signaling pathways linked with inflammation [10,11,12], and is frequently used for intestinal infections [13] (Figure 1). Previous findings based on in vitro studies showed that berberine induces apoptosis in acute lymphoblastic leukemia (ALL) cells via downregulating the MDM2 oncoprotein. [14]. Moreover, berberine’s role as an anticancer agent has also been explained in an earlier study [15]. This article reviews the antitumor activity of berberine on various cancers and assesses the existing suggestion of predicting the possible anticancer effects through modulating the cell signaling pathway.

## 2. Major Possible Mechanisms of Action of Berberine in the Prevention of Cancer

Berberine shows cancer preventive activities through modulating various cell signaling pathways. Previous experimental studies confirmed the anticancer potential of berberine through suppressing metastasis, inhibiting cell proliferation, modulating inflammation, and inducing cell apoptosis, angiogenesis, transcription factors, cell cycle arrest, and other genetic pathways (Table 1 and Figure 1). In addition, activating autophagy, regulating gut microbiota, and enhancing the effects of other antitumor drugs are some of the potential anticancer mechanisms of berberine [16]. By downregulating the expression of proteins linked to metastasis, BBR also prevents tumor cell invasion and metastasis. Furthermore, by decreasing epithelial-mesenchymal transition protein expression, BBR is beneficial in the early stages of cancer development [17].

### 2.1. Antioxidant Potential

Reactive oxygen species (ROS) play a significant role in the cellular signal pathways in normal as well as cancer cells [18]. Moreover, redox homeostasis is controlled by a balanced status between scavenging and ROS production [18,19]. Antioxidants are free radical scavengers that play a significant role in the neutralization of free radicals, thus inhibiting the development and progression of pathogenesis including cancer. Medicinal plants or active compounds of medicinal plants are rich sources of antioxidants, and have the capacity to prevent free radicle formation and inhibit cancer development. Berberine has been noted as a potential therapeutic candidate for liver fibrosis due to its antioxidant and anti-inflammatory activities [20]. Moreover, berberine defends PC-12 cells from oxidative injury via decreasing the ROS level, mitophagy through PI3K/AKT/mTOR signaling pathways, and mitochondrial dysfunction, which advocate a possible therapeutic line for neurotoxic damages and oxidative stress [21]. In addition, native berberine is capable of improving cytotoxic activity better than its derivatives [22]. Berberine inhibited human umbilical vein endothelial cells (HUVECs), and less than 50 µg/mL of berberine inhibited the vascular endothelial growth factor (VEGF) expression to some extent, indicating that berberine suppressed angiogenic action under the condition of little cytotoxicity [23].

### 2.2. Inflammation

It is usually accepted that up to 25% of human cancers are linked to chronic inflammation and to viral and bacterial infections [24]. In addition, cancer-linked inflammation represents the seventh hallmark in the development of cancer [25]. Chronic inflammation leads to major tissue remodeling, loss of tissue structure, and modification of protein self-neutralizing DNA produced by oxidative stress, all of which increase the risk of cancer development [26,27]. Moreover, during tumor development, inflammatory mediators, including ROS, cytokines, and reactive nitrogen species (RNS) derived from tumor-infiltrating immune cells, cause the induction of epigenetic changes in tumor suppressor genes and lead to a pre-malignant state [28]. Pro-inflammatory cytokines and growth factors, including TGF-β and vascular endothelial growth factor (VEGF), then activate several signaling pathways, principally those concerning STAT3 and NF-κB [29].

Medicinal plants or active compounds of medicinal plants play a significant role in the prevention of pathogenesis [30,31] through inhibition of pro-inflammatory cytokines and growth factors. Berberine represses the increased phosphorylation of c-Fos and c-Jun in scratched cancer cells. It has also been noticed that berberine inhibits the activation of NF-*κ*B by inhibiting the degradation of I*κ*B*α* [32]. The antitumor activity of berberine against the triple-negative breast cancer cell line clarifies its mechanism, specifically regarding anti-inflammation. Berberine showed a significant decrease in the proinflammatory cytokines, interleukin and tumor necrosis factor-α. Moreover, berberine efficiently affects both tumor extension and spontaneous metastasis, which provides a novel mechanism related to the inhibition of the inflammasome pathway [33]. The oral administration of berberine has revealed a therapeutic role in chronic ulcerative colitis, including a decrease in intestinal inflammation, prevention of fibrosis, and defense of the intestinal barrier function [34].

### 2.3. Apoptosis

**Figure 2:**
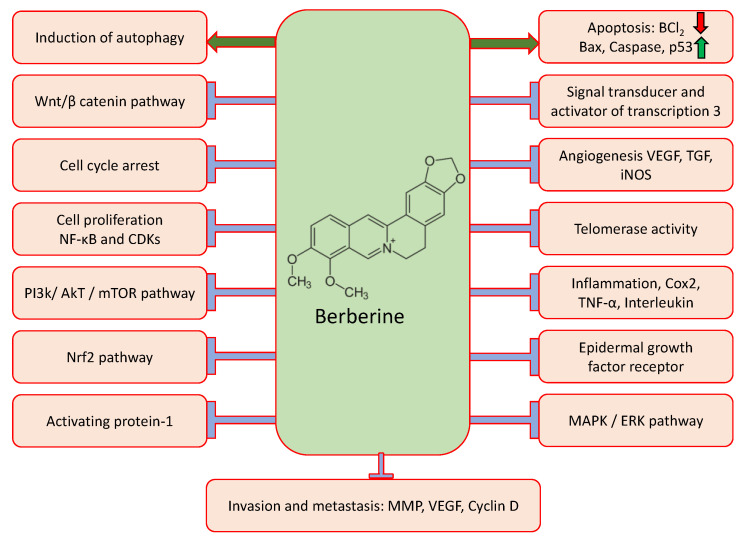
Berberine controls inflammation, angiogenesis, apoptosis, cell cycle, and activities of signaling molecules.

Apoptosis, controlled cell death, is excellently regulated at the gene level by the initiation of DNA damage and results in the effective removal of damaged cells [35]. The Bcl-2 family proteins include pro-apoptotic and anti-apoptotic proteins that act by regulating apoptosis, particularly through the intrinsic pathway, as they exist upstream of the irreversible cellular damage cascade and act chiefly at the mitochondria level [36]. The altered apoptosis process shows a role in cancer development and progression. Therefore, in search of a way to induce apoptosis in cancer cells, berberine may play a significant role in cancer inhibition through induction of apoptosis (Figure 2). Anticancer potentiality of the secondary metabolites of plant extracts is employed via DNA damage and apoptosis induction in cancer cells [37].

Berberine causes activation of mitochondrial apoptosis in liver cancer cells via enhancing the expression of Bax, activation of the caspases 3 and 9 pathway, and cytochrome C release to the cytosol, and inducing autophagic cell death [38]. A study based on breast cancer reported that after treatment of berberine and cisplatin, the cellular cleaved capspase-3 and caspase-9 and pro-apoptotic capase-3 were upregulated, and the downregulation of anti-apoptotic Bcl-2 was noticed [39]. The tamoxifen- and berberine-induced cell growth inhibition was found to be more efficient than that of tamoxifen alone, and combined treatment had greater potential in encouraging the arrest of the G1 phase and inducing apoptosis as compared to tamoxifen alone [40]. Apoptosis induced by berberine in liver cancer cells caused cell cycle arrest at the M/G1 phase and increased the Bax expression [41]. A recent study based on breast cancer reported that a mixture of curcumin and berberine effectively decreases growth in breast cancer cell lines. The combined treatment caused the phosphorylation of c-Jun N-terminal kinase and decreased the phosphorylation of BCL-2 [42].

### 2.4. Autophagy

Autophagy is an evolutionary conserved cellular process where lipids, proteins, and organelles are degraded in lysosomes [43]. Moreover, autophagy is used for the removal of other pathogens [44] and for the engulfment of apoptotic cells [45]. Berberine treatment significantly prevents acute lymphoblastic leukemia cell viability and promotes cell death through induction of autophagy in a dose-dependent way. Moreover, berberine causes autophagic death in leukemia cells via inactivating mTORC1/AKT signaling [46]. Berberine acts as a suppressor of autophagy and inhibits autophagosome formation. Furthermore, berberine treatment obstructs the accumulation of the autophagy-associated proteins, and thus inhibits autophagy [47].

### 2.5. Cell Cycle

Alterations in the cell cycle encourage the development of cancer [48,49]. Induction of cell cycle arrest at various cell cycle checkpoints contributes to various antitumor effects [50]. Cell cycle arrest at the G1 phase, G2/M, or G0/G1 phase is a central event in the inhibition of cancer development and progression. Berberine plays a significant role in the management of cancer. Berberine inhibited cell proliferation in a concentration-dependent way. Moreover, berberine induced cell cycle arrest at the G2/M phase and inhibition of cell proliferation by G2/M phase arrest [51]. The primary mechanisms of repressing the effects of berberine on osteosarcoma cells and normal osteoblasts were investigated. The inhibition was mainly credited to cell cycle arrest at G1 and G2/M, and G1 arrest was dependent on p53. Moreover, apoptosis and induction of G1 arrest were convoyed through a p53-dependent upregulation of pro-apoptotic and p21 genes [52]. Berberine showed a role in cell cycle arrest in cancer as berberine stops human gastric carcinoma cell entrance into the cell cycle in the G0/G1 phase [53,54]. Berberine treatment caused the arrest of the G0/G1 phase, and the outcome of berberine treatment on the cell cycle was time dependent as it brings about in a late cell cycle [55]. Berberine was shown to play a role in the inhibition of the proliferation of multiple myeloma cells and triggered cell cycle arrest [56].

### 2.6. Angiogenesis

During early tumor development and progression, hypoxia activates the transcription of several genes that are important mediators of the angiogenic process, including cVEGF [57]. Mechanically, angiogenic process activation causes the breakdown of the vascular ECM at diverse levels for consequent tube formation and endothelial cell invasion [58]. Inhibition of the angiogenesis process is a key event in the prevention of cancer development and progression. A previous study proved that natural compounds play a significant role in controlling the angiogenesis of tumors by inducing the apoptosis of endothelial cells and preventing angiogenesis-linked cytokines [59].

Berberine’s anti-angiogenic effect has been confirmed through in vitro and in vivo experiments, as berberine showed anti-angiogenic effects via preventing proinflammatory and VEGF, metalloproteinase inhibitor, and interleukin-2 and 6 [23]; berberine also prevented the expression of VEGF [60]. Berberine plays an anti-angiogenic role through inhibition of the capacity of hepatocellular cell carcinoma to stimulate human umbilical vein endothelial cell proliferation, and endothelial tube formation and migration. Moreover, berberine stops the secretion of VEGF from carcinoma and VEGF mRNA expression downregulation [61]. An important finding revealed that a tumor promoter, TPA, meaningfully increased the level of VEGF and fibronectin, whereas the level of fibronectin expression was significantly increased by overexpression of constitutively active (CA)-AKT. Moreover, TPA-caused VEGF and fibronectin expression was reduced by berberine treatment [62].

### 2.7. PI3K/AKT/mTOR Pathway

PI3K/AKT/mammalian target of rapamycin (mTOR) signaling is a vital signaling pathway that plays a significant role in survival, and regulates cell growth, metabolism, and angiogenesis [63,64]. AKT/PI3K/mTOR pathway activation causes cancer development and resistance to anticancer treatments [65]. Inhibition of the AKT/PI3K/mTOR pathway is a vital step toward the prevention of cancer development and progression. In this, berberine plays an important role in cancer management through inhibition of the PI3K/AKT/mTOR pathway [Figure 2]. Additionally, previous preclinical studies of natural compounds are an interesting consideration, and their powerful repressing effects on PI3K/AKT/mTOR signaling pathways have been reported [66,67,68]. Berberine inhibited cancer cell proliferation and showed a role in the induction of apoptosis, arresting the G1 phase of the cell cycle, and MMP depolarization. Berberine also upregulated PTEN dose-dependently, whereas it repressed mTOR, PI3K, Notch1, and Akt protein expression [69]. A gastric cancer-based study reported that berberine induced apoptosis and inhibited PI3K/AKT/mTOR signaling [70]. Phosphorylation levels of Akt and p38 were downregulated by the treatment of berberine. Additionally, the treatment of berberine convoyed by LY294002 or SB203580 meaningfully repressed cell migration and invasion compared to the treatment using berberine alone [71].

### 2.8. Telomerase Activity

Telomeres are focused structures at the ends of linear chromosomes [72,73] and represent repetitive sequences of nucleotides at each terminus of a chromosome. These guard the end of the chromosome to retain genomic stability, and stop degradation and fusion with other chromosomes [74]. Maximum human cancers express high levels of telomerase and short telomeres, while telomerase is absent in most normal somatic tissues [75,76]. Medicinal plants or their bioactive compounds act as telomerase inhibitors and have shown roles in the reduction in telomerase activity in cancer.

Berberine decreased the telomerase activity and level of the colorectal cancer cell line, which in turn decreased the proliferative ability of the cells [55]. A lung cancer-based study reported that berberine has an inhibitory effect on cell proliferation, and this was mediated by the decreased expression of activating enhancer-binding proteins-2α and -2β, which are both needed for hTERT expression. Moreover, in this state, a decreased expression of telomerase was seen [77].

### 2.9. Wnt/β-Catenin Signaling Pathway

The Wnt/β-catenin signaling pathway is a preserved signaling axis contributing to varied physiological processes including apoptosis, proliferation, migration, invasion, and tissue homeostasis [78,79,80]. Altered Wnt/β-catenin signaling pathways showed a role in the development and progression of cancer. Berberine inhibited β-catenin transcriptional activity and reduced anchorage independent growth. After berberine treatment, the active β-catenin level was decreased, connected with an increased expression of E-cadherin. Therefore, berberine and its derivatives have the ability to inhibit β-catenin/Wnt signaling in tumorigenesis [81]. The β-catenin protein level in the cytoplasm and nucleus was decreased with berberine treatment, and this may lead to the inhibition of mRNA expression of β-catenin [82].

### 2.10. Epidermal Growth Factor Receptor

Epidermal growth factor receptor and the EGF family of peptide growth factor play a vital role in the pathogenesis, development, and progression of cancers [83,84]. Interestingly, it was reported that berberine treatment decreased cell proliferation and epidermal growth factor receptor expression levels in the xenograft model. Lastly, these findings indicate that berberine improves Cbl activity, which is subsequent in the downregulation of EGFR expression and the inhibition of proliferation in colon tumor cells [85]. An in vitro-based study reported that expression of prostate specific antigen, in addition to the activation of epidermal growth factor receptor, was inhibited by berberine [86].

### 2.11. Activating Protein-1 (AP-1)

Activating protein-1 is a dimeric transcription factor that classically contains one member from each of the Jun and Fos families [87,88]. Previous experiments based on in vivo and in vitro studies demonstrated that the activity of activating protein-1 is critical for tumorigenesis, as its inhibition by dominant negative c-Jun mutants or activating protein-1 acts as a powerful decoy and prevents the development of numerous tumor cell lines [89,90,91]. Natural compounds play a role in the activating protein-1 pathways in the involvement of pathogenesis, including cancer.

Berberine efficiently targets both the host and the viral factors accountable for cervical cancer development via inhibition of activating protein-1 and hindering of viral oncoproteins’ E7 and E6 expression. Moreover, inhibition of activating protein-1 activity by berberine may be one of the mechanisms responsible for the anti-HPV outcome of berberine [92]. Another study based on the human pulmonary giant cell carcinoma cell line reported that berberine decreased the activity of the activating protein-1 signaling pathway and reduced the binding of transcription factors to the motif of *CCND1* AP-1 [93].

### 2.12. Nuclear Factor Kappa B (NF-κB)

Nuclear factor kappa B (NF-κB) is an important transcription factor that participates in the inflammatory pathway, and several signaling pathways concerned with cancer progression are able to interact with the activation of the NF-κB [94,95]. However, inhibition of NF-κB is an important step towards cancer inhibition. Inflammation-linked cancer can secrete various chemokines and cytokines via NF-κB binding to the promoters of genes [96]. An interesting study based on lung cancer reported that berberine promoted cell morphology change, inhibited proliferation, colony formation, and cell migration, and prompted cell apoptosis. Moreover, berberine inhibited lung cancer cell growth by concurrently targeting NF-κB/COX-2, PI3K/AKT, and cytochrome-c/caspase signaling pathways [77]. In alloxan-induced diabetic mice, renal I kappaB-alpha protein was significantly reduced and the nuclear staining of NF-kappaB p65 was increased in the glomerulus. After berberine treatment, NF-kappaB was decreased, and the reduced degradation of I kappaB-alpha level was partially restored [97].

The role of berberine on irinotecan (CPT-11)-induced apoptosis was examined via the inhibition of NFκB. NF-κB activation was suppressed by berberine treatment in a dose-dependent fashion and improved chemosensitivity to CPT11 through downregulation of NF-κB activation of antiapoptotic genes. Furthermore, berberine inhibits activation of NF-κB and may be used to improve CPT-11-caused apoptosis [98]. The increased levels of pro-inflammatory cytokines (TNF-α, IL-1β, and IL-6), enzymatic antioxidants, non-enzymatic antioxidants, and transcription factor NF-κB were decreased meaningfully by administration of berberine [99].

### 2.13. Nrf2 Pathway

Nrf2 is a cytoprotective transcription factor which showed both a negative and a positive effect on cancer [100,101]. Nrf2 shows vital roles in chemotherapy resistance through stimulating the metabolism of drug or drug efflux [102]. Berberine fails to induce the radiosensitivity in Nrf2-deficient cells, suggesting that Nrf2 is vital for the action of berberine. Berberine suppresses the Nrf2 signaling-related protein expression in HepG2 and Huh7 cells, suggesting that berberine supports radiosensitivity through suppressing the Nrf2 signaling pathway in hepatocellular carcinoma cells. Additionally, an experiment using xenografts in nude mice indicated that berberine enhances the inhibitory growth effect of radiation in an Nrf2-dependent way [103].

### 2.14. Signal Transducer and Activator of Transcription 3 (STAT3)

STAT1, STAT2, STAT3, STAT4, STAT5, and STAT6 are family members of STAT proteins which are imperative transducers of several cytokines and growth factors [104]. STAT3 is the most common, and is overexpressed or constitutively activated in approximately seventy percent of solid and hematological tumors [105]. The effect of berberine on the growth and tumorigenicity of nasopharyngeal carcinoma, and its connection to STAT3 signaling, was evaluated. The results confirmed that berberine effectively inhibited tumorigenicity and growth, and inhibited both constitutive and interleukin-6-induced signal transduction and activator of transcription 3 activations. Inhibition of signal transduction and activator of transcription 3 activations by berberine induced the response of apoptotic and growth inhibition [106]. Berberine prevents the expression of CDC6 and proliferation in human keratinocytes through regulating the JAK–STAT3 signaling pathway [107]. Other bioactives, such as curcumin and epigallocatechin-3-gallate, also showed the inhibition of the STAT3 signaling pathway [108].

### 2.15. MAPK/ERK Pathway

Ginsenoside and berberine induced apoptosis through inducing the expression of apoptosis-associated protein Bax, and preventing the expression of survivin and anti-apoptotic protein. Ginsenoside and berberine combined treatment played a role through the MAPK/ERK pathway [109]. Moreover, berberine prevented the metastatic potential of melanoma cells via a reduction in ERK activity, and the protein levels of cyclooxygenase-*2* by a berberine-caused AMPK activation [110]. Berberine suppressed human gastric cancer cell growth in vivo and in vitro via inducing cytostatic autophagy through the inhibition of MAPK/mTOR/p70S6K [111].

**Table 1:** Anticancer potential of berberine through modulating cell signaling pathways.

Cell lines	Genes/Pathways	Effects	Mechanism	Refs.
MDA-MB-231	Proinflammatory cytokines	Inhibited proinflammatory cytokines	Berberine meaningly decreased the increased expression of IL-6 and TNF-α. Furthermore, berberine inhibited NF-κB activation through inhibiting the degradation of IκBα.	[32]
MDA-MB-231	Proinflammatory cytokines	Inhibited proinflammatory cytokines	Berberine showed a significant decrease in the proinflammatory cytokines, interleukin, and tumor necrosis factor-α	[33]
MHCC97-L and HepG2	Apoptosis	Induced apoptosis	Berberine causes activation of mitochondrial apoptosis via enhancing expression of bax, activation of the caspases	[38]
MCF-7	Apoptosis	Induced apoptosis	Berberine treatment upregulates caspase 3 and 9 and downregulates the anti-apoptotic Bcl-2	[39]
Huh7 and WRL68	Apoptosis	Induced apoptosis	Berberine caused cell cycle arrest and increased the Bax expression	[41]
MCF-7/ADR cells	Autophagy	Inhibited autophagy	Berberine reverses doxorubicin resistance through preventing autophagy	[47]
U2OS, Saos-2 and HOS		Cell cycle arrest	The inhibition was mainly credited to cell cycle arrest at G1 and G2/M, and G1 arrest was dependent on p53	[52]
LoVo		Cell cycle arrest	Berberine-treatment displayed accumulation of cells in the G2/M phase	[53]
HCT116		Cell cycle arrest	G_0_/G_1_ phase arrest	[55]
HepG-2 and HUVECs		Inhibited vascular endothelial growth factor	Berberine downregulates expression VEGF mRNA and prevents secretion of vascular endothelial growth factor	[61]
SW480		Inhibited PI3K/Akt/mTOR	Berberine upregulated PTEN, and inhibited proteins of Akt, PI3K, and mTOR	[69]
SGC-7901 and BGC-823		Inhibited PI3K/AKT/mTOR	Berberine meaningfully repressed the PI3K/AKT/mTOR	[70]
HCT 116		Inhibited telomerase/TERT	Telomerase activity was decreased by berberine	[55]
A549		Targeted AP-2/hTERT,	Cancer cell growth was inhibited by berberine, which also directed AP-2/hTERT	[77]
HCT116		Inhibited Wnt/β-catenin	Berberine prevented Wnt/β-catenin signaling	[81]
PC-3 and LnCaP		Inhibited epidermal growth factor receptor	Proliferation of cancer cancer cells was inhibited by berberine via cell cycle arrest, and/or apoptosis by EGFR signaling pathway inactivation	[86]
PG		Inhibited Activator protein 1	Berberine repressed the c-Jun expression and reduced the transcription factors binding to the CCND1 AP-1 motif	[93]
HCT 116		Inhibited NF-κB	Berberine inhibits NF-κB activation	[98]
HepG2 and Huh7 cells		Suppressed Nrf2	Berberine supports radiosensitivity via Nrf2 signaling pathway suppression	[103]
C666-1		Inhibited signal transducer and activator of transcription 3	Berberine inhibited IL-6-induced STAT3 activation	[106]
A375		AMPK	Berberine induced AMPK activation	[110]
BGC-823		mTOR/MAPK	Berberine inhibited mTOR/MAPK/p70S6K	[111]

## 3. Role of Berberine in Management of Various Types of Cancer

Medicinal plants or their active compounds act as inhibitors of cancer development through the modulation of cell signaling molecules. Berberine plays a vital role in the management of numerous types of cancer. The role of berberine in cancer prevention has been noticed in several cancers through modulating cell signaling pathways (Table 2). The specific role of berberine in the management of different cancers is discussed here.

### 3.1. Prostate Cancer

The molecular mechanism of berberine in the management of prostate cancer was investigated. Migratory and invasive capacities of metastatic prostate cancer cells were inhibited by berberine treatment. Moreover, the berberine inhibitory effects lead to significant repression of mesenchymal genes that play a role in the regulation of the developmental EMT [112]. Berberine reduced the androgen receptor transcriptional activity and caused androgen receptor protein degradation. Remarkably, it was seen that androgen receptor splice variants were more vulnerable to berberine-caused degradation. The growth of LNCaP xenografts in nude mice was prevented through berberine treatment, and androgen receptor expression was decreased in the tumors [113]. Another study based on prostate cancer reported that inhibition of 22Rv1prostae cancer cells’ proliferation and cellular testosterone formation was decreased by berberine in a dose-dependent way. The aldo-keto reductase family 1 member C3 enzyme activity was inhibited by berberine [114]. The effect of berberine on the radiosensitivity of prostate cancer was investigated and findings revealed that berberine increased xenografts and radiosensitivity of prostate cancer cells in a dose-dependent way, and this was connected with the prevention of hypoxia inducible factor-1α (HIF-1α) and vascular endothelial growth factor expression [115]. The proliferation of prostate cancer PC3 and RM-1 cells was notably inhibited by berberine treatment, and inhibitory effects on cancer cells were improved in a time-and concentration-dependent manner [116]. Meeran and colleagues revealed that reactive oxygen species regulate berberine-mediated cell death in human prostate cancer cells, and therefore suggested that berberine might be considered for further studies as a promising therapeutic candidate for prostate cancer [117]. In 2017, Li and colleagues carried out metabolic characterization and pathway analysis, showing that berberine protects against prostate cancer. The study concluded that berberine could be used to treat prostate cancer by regulating purine metabolism, linoleic acid metabolism, retinol metabolism, proline and arginine metabolism, retinoate biosynthesis, and spermine biosynthesis [118]. Through the induction of apoptosis in prostate cancer cells, berberine reduces the growth of p53-dependent prostate cancer cells [119].

### 3.2. Urinary Bladder Cancer

Cytotoxic effects against bladder cancer cell lines were noted following berberine treatment through in vivo and in vitro experiments. Induced DNA replication defects and arrest of the cell cycle, subsequent in apoptosis and dependent on p53 status, were observed following berberine treatment. Mechanistically, antitumor effects on bladder cancer cells were noted following treatment of berberine, exerted by inhibiting Janus kinase 1- *STAT 3* signaling via miR-17-5p upregulation. It binds to the signal transducer and activator of transcription 3 and 3’UTR of JAK1, and downregulates their expression [120]. Transfection of hpa-siRNA and treatment with berberine reduced the invasion and migration of cancer cells. Consequently, the invasion and metastasis of bladder cancer cells were inhibited by berberine, probably through delaying the expression of heparinase, and therefore may be used clinically to decrease the recurrence of bladder cancer [121]. By comparison, berberine repressed the viability of cancer cells such as T24 and BIU-87 cells in a dose- and time-dependent fashion. In a dose-dependent manner, berberine induced apoptosis and promoted cell cycle arrest at G0/G1. Berberine treatment decreased the H-Ras and c-fos mRNA and protein expressions in a dose- and time-dependent fashion. Furthermore, berberine induces cell cycle arrest and apoptosis and inhibits cell proliferation [122]. In addition, arylamine-N-acetyltransferase activity was inhibited by berberine in human bladder tumor cells in a dose-dependent way [123]. Inducing apoptosis in human bladder tumor cells with two telomerase-targeting complexes of jatrorrhizine and berberine derivatives showed that the combination may be a novel anticancer drug candidate [124]. By inhibiting the PI3K/Akt pathway and downregulating Rad51 expression, BER can increase GEM-induced cytotoxicity in BC, which may represent a novel therapeutic target for BC treatment. By inhibiting the PI3K/Akt pathway and downregulating Rad51 expression, BER can increase GEM-induced cytotoxicity in BC, which may represent a novel therapeutic target for BC treatment. By inhibiting the PI3K/Akt pathway and downregulating Rad51 expression, BER can increase GEM-induced cytotoxicity in BC, which may represent a novel therapeutic target for BC treatment. By inhibiting the PI3K/Akt pathway and downregulating Rad51 expression, BER can increase GEM-induced cytotoxicity in BC, which may represent a novel therapeutic target for BC treatment [125].

### 3.3. Renal Cell Carcinoma

The effects of berberine related to photodynamic therapy in renal carcinoma cell lines were evaluated. Increased cytotoxicity in a concentration- and time-dependent manner was noted by cellular viability assay. Furthermore, after treatment with berberine, a high phototoxicity effect was seen, with less than 20% of viable cells. Moreover, three target genes of anticancer drugs were expressed, namely, VEGF-D and telomerase reverse transcriptase (TERT) presented low expression, and overexpression of Polo Like Kinase 3 (Plk-3) was noticed after treatment with berberine [126]. By downregulating Mcl-1 and c-FLIP through ROS, berberine has been reported to promote TRAIL-induced apoptosis in human renal cancer cells [127]. In a study on renal cancer, berberine raised the levels of autophagy and reactive oxygen species in human renal tubular epithelial cells derived from the normal kidney HK-2 cell line, in addition to human cell lines ACHN and 786-O cell line. By activating caspase 3, berberine caused these cells to undergo apoptosis [128].

### 3.4. Liver Cancer

An in vitro-based finding suggested that berberine inhibited the proliferation of cancer cells. It inhibits solute carrier family 1 member 5 (SLC1A5), Na^+^ dependent transporter, and, in this way, berberine suppresses the glutamine uptake. Moreover, berberine suppresses SLC1A5, Na^+^ dependent transporter expression through preventing c-Myc. Additionally, the growth of tumor xenografts is suppressed by berberine and the SLC1A5 expression and c-Myc [129]. Berberine inhibits cancer cell viability, and berberine may control proliferation through the regulation of numerous tumorigenesis-associated genes and protein expression. The expression of tumor suppressor genes was upregulated, and the expression of pituitary tumor transforming gene 1 and E2F transcription factor 1 was downregulated [130]. Berberine arrests the G0/G1 phase cell cycle of cancer cells, deactivates the Akt pathway, and suppresses the S-phase kinase-associated protein 2 expressions. In conclusion, berberine endorses the expression of p27^Kip1^ and cyclin-dependent kinase (CDKIs p21^Cip1^) and additionally induces G0/G1 phase cell cycle arrest of hepatocellular carcinoma [131]. Another interesting study reported that berberine decreases the viability of cancer cells, in a dose- and time-dependent way, and the number of apoptotic cells increased accordingly. Translocation of apoptosis-inducing factors between the mitochondria and the nucleus was induced by berberine and decreased the protein expression levels of cyclooxygenase-2 and cytosolic phospholipase A2. Moreover, in mice, berberine decreased the volume and the weight of tumors in a transplanted tumor model [132]. In liver cancer cells such as HepG2 and MHCC97-L, berberine may also cause autophagic cell death through activation of Beclin-1 and inhibition of the mTOR-signaling pathway by downregulating Akt activity and upregulating P38 MAPK signaling [38]. Berberine, a natural plant alkaloid, synergistically sensitizes human liver cancer cells to sorafenib [133].

### 3.5. Colorectal Cancer

Cell viability of colorectal cancer was inhibited by the treatment with berberine through inducing apoptosis levels. Moreover, long non-coding RNAs CASC2 was upregulated in cells via berberine, and knockdown of long non-coding RNAs CASC2 overturned the berberine-encouraged apoptosis. Furthermore, Bcl-2 was repressed by berberine treatment and long non-coding RNAs CASC2, encouraging the pro-apoptotic effects [134]. By comparison, Par3 and E-cadherin were significantly decreased in tumor tissues, and miR-429 enhancement was observed in tumor tissues. With berberine and evodiamine treatment, the level of miR-429 was decreased in the tumor tissue [135]. Moreover, berberine treatment induced apoptosis, repressed colon cancer cell viability, and activated caspase-3 activity in the colon carcinoma cell line. The expression of miR-21 was inhibited and ITGβ4 and PDCD4 protein expression was promoted in the cancer cell line by berberine. Moreover, high miR-21 expression decreases the anticancer effects of berberine on apoptosis rate, cell viability, and caspase-3 activity of the cancer cell line [136]. In another study, berberine was reported to hinder the COX-2/PGE2-mediated JAK2/STAT3 signaling pathway in colorectal cancer cell invasion and metastasis [137]. By downregulating GRP78, berberine prevents colorectal cancer cells from proliferating and migrating [138].

### 3.6. Pancreatic Cancer

Ming et al. (2014) explained that berberine showed anticancer activity, and inhibited proliferation of cancer cells and suspension of progression of its cell cycle of G1 and reduced cell growth. Berberine also inhibited DNA synthesis and mitogenic signaling via an AMPK-independent mechanism at higher concentrations. Finally, metformin and berberine constrain mitogenic signaling in cancer cells [139]. A novel study was performed based on berberine to evaluate the anticancer activity. Initiation of apoptosis and cell cycle capture was noted by the treatment of berberine in a dose-dependent manner. The G1 phase of cancer cells increased by 10% after berberine treatment and the G1 phase was increased by 2%. Moreover, gemcitabine showed antiproliferation effects via S-phase arrest and berberine prevented proliferation via causing G1-phase arrest. Remarkably, berberine showed a greater apoptotic effect than gemcitabine in cancer cells [140]. After berberine treatment, it was noticed that berberine showed privileged selectivity towards cancer cells as compared to normal ones. Furthermore, ingenuity pathway and expression profiling findings revealed that the cytotoxicity of berberine was convoyed with G1/S and G2/M cell cycle checkpoint regulation and activation of P53 signaling pathways. The activation of these signaling pathways can be described by the fact that berberine induces DNA strand breaks and intercalates DNA [141]. The therapeutic ability of berberine on pancreatic cancer cells was investigated through the cell metabolomics method. The important inhibitory role of berberine in pancreatic cancer cell viability and metastasis was observed. Berberine also greatly dysregulated the energy metabolism of cancer cells, and the mitochondria of these cells were evidently damaged after treatment with berberine. Remarkably, transportation and citrate metabolism in mitochondria were meaningfully influenced by berberine [142]. The ability of berberine and chemically altered berberines to prevent the proliferation of pancreatic cancer cells has been reported by Abrams and colleagues [143]. Berberine inhibited pancreatic cancer cell viability and metastasis by controlling citrate metabolism [142]. BBR caused inhibitory effects in pancreatic cancer cells on the expression of Rad51 and the upregulation of PARP expression compared with control pancreatic cancer cells [144].

### 3.7. Gastric Cancer

Berberine seems to cause its anticancer activity by encouraging ROS production and preventing cell migration through inhibition of matrix metalloproteinases-1, -2, and -9 gene expression [145]. Curcumin, quercetin, and berberine efficiently downregulated survivin expression, pSTAT3 levels, and gastric cancer cell viability in a dose-dependent manner. Berberine was more effective in inhibiting survivin expression as compared to other natural compounds. 5-fluorouracil in combination with berberine or curcumin caused a synergistic inhibition of STAT3 and survivin level, resulting in enhanced cell death in gastric cancer cells [146]. Berberine efficiently improves the activity of cetuximab and erlotinib based on in vitro and in vivo studies. Moreover, berberine induces apoptosis and was noticed to inhibit growth in gastric cancer cell lines. Such things were related to the inhibition of activation of EGFR signaling [147]. Berberine can inhibit gastric cancer cell migration, proliferation, invasion, and suppression of gastric tumor growth. The anti-gastric cancer mechanism of berberine may involve the HNF4α- AMPK-WNT5A signaling pathway and HNF4α is a key molecule target [148]. Berberine concentration-dependently downregulated the multi-drug resistance-1 protein levels in the BGC-823/DDP and SGC7901/DDP cells and multidrug resistance-associated protein 1. Remarkably, the cell apoptosis of BGC-823/DDP and SGC-7901/DDP cells was significantly increased by co-treatment with berberine and cisplatin. The results from animals also showed that berberine treatment sensitized SGC-7901/DDP cells to DDP in vivo [70]. Berberine has the potential to influence cancer-related pathways by regulating circRNA expression and their corresponding target genes in GC cells [149].

Berberine time- and dose-dependently inhibited the proliferation of cancer cells. It also suppressed tumorigenesis in nude mice xenografted with MGC 803 cells. Moreover, berberine reduced interleukin-8 secretion by in vitro and in vivo systems. Berberine may be an effective and safe drug option for treating gastric cancer through modulation of the MAPK signaling pathways [150]. The berberine-induced Apoptosis of human gastric cancer cells is associated with AKT signaling [151]. In addition, berberine and curcumin have been found to target survivin and STAT3 in gastric cancer cells [146].

### 3.8. Bile Duct Cancer

Berberine has been also investigated for its growth inhibitory effects with the proper mechanism on cholangiocarcinoma cell lines. The growth of cholangiocarcinoma cell lines was significantly inhibited in a dose- and time-dependent manner by berberine. The inhibition was credited to cell cycle arrest at the G1 phase via reduction of cyclin E and cyclin D1 [152]. A parallel study based on human cholangiocarcinoma cells reported that berberine dose-dependently decreased cell viability and caused cell death induction, which was related to an increase in the arrest of the G1 phase. Moreover, G1 cell cycle arrest induced by berberine was arbitrated via the cyclin-dependent kinase inhibitors as the protein expression increased [153]. A study proved the therapeutic efficacy of cucurbitacin B loaded phospholipid complex modified with berberine hydrochloride for cholangiocarcinoma [154]. Berberine hindered the growth and induced apoptosis in human cholangiocarcinoma QBC939 cells [153].

### 3.9. Esophageal Cancer

The effect of berberine on esophageal cancer cells and its mechanism of action were examined. Growth inhibition of cancer cells such as KYSE-70 and SKGT4 cells was noticed through berberine treatment in a time- and dose-dependent fashion. In KYSE-70 cells, berberine treatment with fifty μmol/L showed the G_2_/M phase cells were significantly more numerous as compared to the control group [155]. The migration rate of the cells was decreased after treatment of berberine with different concentrations, and the chemokine receptors’ expression levels involved in the metastasis and migration of cancer cells were decreased [156]. Berberine at lower concentrations considerably radio-sensitized esophageal carcinoma cells. Berberine pretreatment led to a significant downregulation of RAD51 in cells of esophageal squamous cell carcinomas [157]. Galangin and berberine’s combined anticancer effects were reported on esophageal carcinoma cells by inducing apoptosis and inhibiting proliferation [158]. Coptidis Rhizoma and berberine’s ability to prevent human esophageal cancer cell lines from proliferating was proven by Lizuka and colleagues [159].

### 3.10. Lung Cancer

Based on in vitro studies, non-small cell lung cancer cell proliferation and colony formation were decreased by berberine treatment. It also inhibited cancer growth in subcutaneously transplanted tumor lung models. Furthermore, 646 genes were differentially expressed upon administration of berberine. Berberine showed a role in the downregulation of the expression of RRM2, RRM1, POLE2, and LIG1, which involve DNA replication and repair [160]. Berberine suppressed growth and induced apoptosis in non-small cell lung cancer cells [161]. Berberine and cinnamaldehyde decrease mouse vulnerability to urethane-induced lung carcinogenesis. In vitro studies, cinnamaldehyde and berberine together induced cell apoptosis, and prevented cell proliferation, wound healing and autophagy, AMPK upregulation, and downregulation of AQP-1. Moreover, the cinnamaldehyde/berberine combination prevented A549 cell substance permeability and decreased intracellular concentrations of ATP [162]. Berberine induced apoptosis, inhibited cell proliferation, and suppressed tumor spheroid formation in the lung cancer cell lines. Additionally, berberine promoted signal transduction and activation of transcription 3 degradations via enhancing ubiquitination. It was able to inhibit doxorubicin-mediated signal transducer and activator of transcription 3 activations [163]. Treatment of berberine hydrochloride inhibited the vascular endothelial growth factor expression and transcription factor AP-1 and nuclear factor κB proteins. Moreover, it was found that berberine hydrochloride treatment may inhibit proliferation, enhance apoptosis, and increase the cytotoxicity of different cancer cells [164]. The role of autophagic cell death in the combined radiation and berberine therapy was shown in the lung cancer’s synergistic tumor-killing effect [165]. Berberine inhibits the invasion of human lung cancer cells by reducing the levels of matrix metalloproteinase-2 and urokinase-plasminogen activator [166].

### 3.11. Oral Cancer

Berberine showed an anticancer role through G0/G1-phase arrest induction, Ca^2+^ production, and induction of reactive oxygen species [167]. Induction of apoptosis in KB cells by berberine was observed, and was partially reversed by incorporation of prostaglandin E2. Berberine treatment inhibited Mcl-1 and cyclooxygenase-2 expression dose-dependently [168]. Viability of SCC-4 cells was reduced by berberine, which was introduced by the ROS generation, through an increase in cytosolic Ca^2+^ concentration. Berberine also caused the induction of apoptosis linked with a decrease in the mitochondrial membrane potential [169]. Human HSC-3 oral cancer cells are subjected to apoptosis by berberine when the death receptor-mediated and mitochondrial pathways are simultaneously activated [167]. Berberine modulates apoptosis in oral cancer cells by inhibiting the expression of cycloxygenase-2 and Mcl-1 [168]. Berberine-induced cell death of KB oral cancer cells was mediated by both intrinsic mitochondrial-dependent and extrinsic death receptor-dependent apoptotic signaling pathways. Moreover, berberine-induced upregulation of FasL was revealed to be mediated by the p38 MAPK signaling pathway [170].

### 3.12. Cervix Cancer

Berberine precisely downregulates the expression of oncogenic c-Fos and the treatment by this alkaloid caused suppression of E6 and E7 levels and a concomitant increase in Rb and p53 expression in both used cell types. Moreover, berberine repressed the telomerase protein expression, which translated into inhibition of cervical cancer cell growth [93]. Berberine hydrochloride showed a role in cervix cancer by inhibiting the proliferation and inducing apoptosis, potentially via a decrease in Bcl-2 and the enhancement in p53 [171]. By comparison, berberine showed a role in the inhibition of cancer cell viability, invasion, and migration, in addition to inducing apoptosis via Bax and enhancing caspase-3 expression, and decreasing the expression of Bcl-2 [172]. Berberine effectively damaged cervix cancer cells and HeLa cells under hypoxic and low-glucose conditions and decreased the number of colony counts. Moreover, low doses of berberine decreased the level of HIF-1α and phospho-PI3K under nutrient-deprived conditions [173]. Another finding reported that berberine meaningfully decreased the Bcl-2/Bax ratio. The upregulation of Fas, FasL, tumor necrosis factor receptor (TNFR) associated factor 1, and TNF-alpha suggested the involvement of the death receptor pathway in the process of berberine-caused apoptosis. Furthermore, berberine showed a role in the increment of the expression of p53 in cancer-treated cells [174]. Berberine inhibits HPV transcription and downstream signaling in cervical cancer cells, causing growth arrest and apoptosis through modulation of AP-1 activity [92]. Berberine increased GADD153 expression via inducing reactive oxygen species production, and then led to mitochondria dysfunction followed by activating caspase-3 and cytochrome C release, which then resulted in cervical cancer cell apoptosis [175]. In human cervical cancer Ca Ski cells, GADD153 mediates berberine-induced apoptosis [176].

### 3.13. Endometrium Cancer

Berberine was found to inhibit the growth, invasion, migration, and metastasis of endometrial cancer cells based on in vitro and in vivo findings. Through miR-101/COX-2, berberine inhibits the growth and metastasis of endometrial cancer cells. Berberine was found to be capable of suppressing tumors through cyclooxygenase-2/prostaglandin E2 signaling pathways. Transcription of miR-101 was enhanced by berberine through activator protein 1 in order to modify the transcription of COX-2 in endometrial cancer cells [177].

In a recent study, the potential cytotoxic and antimetastatic effects of berberine on gynecological cancers with drug-associated resistance were noticed [178]. Berberine inhibited the proliferation, invasion and migration of human endometrial stromal cells via downregulating the expression of miR-429 [179].

### 3.14. Ovarian Cancer

The proliferation of ovarian cancer cells is considerably inhibited by berberine in a dose- and time-dependent way, and apoptosis is induced, probably via downregulation of anti-apoptotic genes survival and BCL-2, and pro-apoptotic gene BAX upregulation. A synergistic anticancer effect was also noted when given with cisplatin, against ovarian cancer cells [180]. Berberine, in a dose- and time-dependent manner, suggestively inhibited the proliferation of primary ovarian cancer cells and OVCAR3 cells. The combined treatment of berberine and cisplatin showed a powerful inhibitory effect on the growth of cancer cells and G0/G1 cell cycle arrest induction [181]. By comparison, it was noticed that DDP/A2780 cells, when incubated with berberine in combination with cisplatin, showed significantly lower survival as compared to the control group. Berberine involvement also improved cisplatin-induced G0/G1 cell cycle arrest and apoptosis [182].

A recent study was performed based on berberine to evaluate the anticancer effects and its molecular mechanisms. It was reported that motility and invasiveness of ovarian cancer cells were reduced by berberine treatment. Moreover, berberine depleted both ErbB2 and EGFR, and suppressed the activation of ErbB2 and EGFR and downstream targets cyclin D1, MMPs, and vascular endothelial growth factor [183]. Two human epithelial ovarian carcinoma cell lines, SKOV-3 and OVCAR-3, exhibited a dose-dependent decrease in their growth rate after the treatment of berberine. Finally, a study concluded that berberine treatment can inhibit proliferation via cell cycle arrest in cancer cells [184]. Berberine has antitumor properties in human ovarian cancer cells [180].

### 3.15. Breast Cancer

A recent report based on in vitro study of breast cancer demonstrated that berberine synergistically increases the inhibitory effect of doxorubicin on tumor cell proliferation. Therefore, the results showed that berberine can reverse multidrug resistance by inhibiting the efflux function of ATP-binding cassette transporters and downregulation of their expression levels [185]. Berberine combined with emodin meaningly inhibited Salt-inducible kinases 3 activity, leading to increased cell cycle arrest, reduced cell growth, and induce apoptosis in breast cancer cells, whereas these effects were not noticed in the nonmalignant breast epithelial cell line. Furthermore, combination treatments induced apoptosis and G0/G1 phase cell cycle arrest [186]. Cell death induction and cell growth inhibition were noticed at higher doses, including more than 10 μM of berberine treatment in breast cancer cells, due to the p53 upregulation under the nucleolar stress response caused by a noteworthy accumulation of berberine in the nucleoli [187].

It has been observed that berberine and exercise co-treatment meaningfully slowed the progression of breast cancer in tumor-bearing mice. Moreover, the synergistic effect on the level of short chain fatty acids increased. Study results suggest that the synergistic treatment of berberine and exercise improves the immune system, activates the mitochondrial apoptosis pathway and Fas death receptor apoptosis pathway, and regulates intestinal microbial metabolites [188]. Berberine treatment with 50 mg/kg body weight applied to breast tumor-bearing rats was found to be powerful against 7, 12-dimethylbenz[a]anthracene-induced mammary carcinoma. The increased levels of pro-inflammatory cytokines, lipid peroxide, enzymatic antioxidants, non-enzymatic antioxidants, and transcription factor NF-κB were decreased clear by the treatment of berberine [96]. Moreover, berberine inhibits the growth of Anoikis-resistant MCF-7 and MDA-MB-231 breast cancer cell lines through inducing cell cycle arrest [189]. In 2008, the manner in which berberine affects the human breast cancer cell lines MCF-7 and MDA-MB-231 was examined. Both cell lines responded to berberine treatment with a time-dependent reduction in proliferation at different concentrations, including 20 M for MCF-7 cells and 10 M for MDA-MB-231 cells. When compared to the corresponding controls, apoptosis increased in both cell lines, by 12% in MDA-MB-231 cells and 31% in MCF-7 cells, according to annexin V staining. These findings show that berberine treatment inhibits the growth of MDA-MB-231 and MCF-7 cells [190]. The mechanisms underlying AMPK activation on doxorubicin (DOX) chemosensitivity, in addition to the chemosensitive effect of various dosages of berberine on drug-resistant human breast cancer MCF-7/MDR cells, were investigated in vitro and in vivo. The outcomes demonstrated that berberine could, through dose-orchestrated administration, overcome DOX resistance. The AMPK-HIF-1-P-gp pathway of low-dose berberine can improve DOX sensitivity in drug-resistant breast cancer cells; furthermore, high-dose berberine alone, independently of HIF-1 expression, directly induces apoptosis through the AMPK-p53 pathway [191].

### 3.16. Thyroid Cancer

After berberine treatment, both TPC1 and 8505C cell lines showed a dose-dependent decrease in growth rate. Moreover, cancer cell line 8505C displayed increased levels of apoptosis, and berberine treatment induced a slight upregulation of p-27 in 8505c cancer cells, but comparatively high upregulation of p-27 in TPC1 cancer cells [192]. Berberine showed a role in the inhibition of the proliferation of different cancer cell lines of thyroid carcinoma cells in a dose- and time-dependent fashion as compared to the control group. Berberine also induces significant mitochondrial apoptosis, inhibitive migration, and G0/G1 cell cycle arrest in thyroid carcinoma cells [18]. Berberine could modulate PI3K-AKT and MAPK signaling pathways in thyroid carcinoma cells, which leads to mitochondrial apoptosis, G0/G1 cell cycle arrest and suppressive migration [193].

Ret Proto-oncogene (RET) expression was suppressed by berberine by more than 90% in medullary thyroid carcinoma cells at a concentration of 2.5 μg/mL. The downregulation of RET with berberine further inhibited cell proliferation [194]. Thyroid cancer cell lines 8505C and TPC1 were inhibited by berberine [192]. Delayed luminescence was used to track the programmed cell death caused by berberine in thyroid cancer cells. Berberine can stop the cell cycle and activate the apoptotic pathway, as evidenced by deoxyribonucleic acid fragmentation, caspase-3 cleavage, and p53 and p27 protein overexpression [195].

### 3.17. Lymphoma

A recent result indicated that the expression of CD47 is downregulated by berberine in diffuse large B-cell lymphoma at the transcriptional level via suppressing c-myc expression. Moreover, berberine-induced CD47 inhibition improved the macrophages’ phagocytosis, thus eliminating diffused large B-cell lymphoma cells based on in vitro and in vivo studies [196]. The cell proliferation in the primary effusion lymphoma cell lines was inhibited by the treatment of berberine. Moreover, treatment with berberine significantly inhibited the invasion and growth of primary effusion lymphoma cells [197]. Berberine, according to Ren and colleagues, has antitumor activity in diffuse large B-cell lymphoma by modulating the c-myc/CD47 axis [196]. It is now known that Kaposi sarcoma-associated herpes virus/human herpes virus-8 (KSHV/HHV-8) is the tumor that causes primary effusion lymphoma (PEL), and nuclear factor (NF)-B activation is essential for the survival and expansion of PEL cells. Berberine has an antitumor effect against primary effusion lymphoma, according to Goto et al., by inhibiting the NF-B pathway [197].

### 3.18. Osteosarcoma

A human osteosarcoma-based study reported that induction of the G2/M phase, in addition to apoptosis and cell cycle arrest, was observed following berberine treatment. Berberine significantly decreased the mRNA and protein expressions of Rad51, and inhibited the invasive capability, decreased vimentin protein, and increased E-cadherin [198]. Based on in vitro and in vivo findings, it was reported that expression of caspase-1 and its downstream target Interleukin-1β (IL-1β) was higher in osteosarcoma cells as compared to normal cells. Additionally, the administration of berberine is capable of inhibiting the growth of tumor cells and decreasing the expression of IL-1β and caspase-1 [199]. Berberine reduced colony formation, cell viability, wound healing ability, and migration of osteosarcoma cells. Similarly, the expression of matrix metalloproteinase 2 was significantly reduced by berberine, suggesting its inhibitory action on the matrix metalloproteinases, which are vital for the invasion of cancer cells [200]. Combination treatment of berberine and cisplatin enhanced the inhibition of cell migration and invasion. In addition, combination treatment induced apoptosis and cell cycle arrest in the G0/G1 phase [201]. Cell apoptosis was examined using flow cytometry and a DNA ladder assay, and the impact of berberine on cell viability was assessed using a 3 (4,5-dimethylthiazol-2-yl) 2,5-diphenyltetrazolium bromide assay. In MG-63 cells, DNA damage was identified using H2AX focus formation.

DNA fragmentation analysis and flow cytometry revealed that berberine significantly increased apoptosis in MG-63 cells in a concentration- and time-dependent manner. Additionally, when compared to the negative control, berberine significantly increased DNA damage in a concentration- and time-dependent manner. Therefore, human osteosarcoma cell line MG63 undergoes apoptosis and DNA damage in response to berberine [202].

### 3.19. Leukemia

Berberine treatment suggestively inhibits acute lymphoblastic leukemia cell viability and encourages cell death through inducing autophagy in a dose-dependent fashion. Furthermore, berberine suggestively improves the aggressive pathological condition in acute lymphoblastic leukemia xenograft mice. Berberine also induces autophagic death through inactivating the mTORC1/AKT signaling pathway [46]. Examination of treated cells showed that berberine decreased Bcl-2 and ROR1 levels. Remarkably, berberine could decrease the expression of miR-21 in comparison to the untreated group [203]. Berberine prompted mitochondrial dysfunction and abnormal cellular energetic metabolism. Cytotoxic/cytostatic action of berberine at 10–30 μM might be mediated, at least in part, via berberine-induced impairment of oxidative phosphorylation and the related increment in oxidative damage [204].

Berberine reduced acute lymphoblastic leukemia cell viability and induced apoptosis. Berberine-induced cell apoptosis was reduced via inhibition of XIAP that was regulated by PIM-2 and berberine treatment induced an enhancement of miR-24-3p [205]. Moreover, berberine-induced G2/M-phase arrest in tested cell lines was convoyed by increased levels of 14-3-3 sigma. In addition, berberine induced G2/M arrest through the promotion of Wee1 and inhibition of cyclin B1 [206]. By comparison, it was reported that berberine-induced apoptosis is related to nucleophosmin/B23 downregulation and telomerase activity. Based on the findings, it was suggested that nucleophosmin/B23 may play a significant role in the control of the cellular response to induction of apoptosis [207]. Berberine’s in vivo effects on WEHI-3 leukemia cells were investigated. The findings revealed that Mac-3 and CD11b markers were decreased, indicating that the precursors to macrophages and granulocytes were prevented from differentiating [208] (Yu et al., 2007).

In promyelocytic leukemia HL-60 cells, berberine showed the ability to cause morphological changes and internucleosomal DNA fragmentation, which are signs of apoptosis. The findings suggest that the berberine-induced apoptosis in HL-60 cells may be caused by various critical cellular processes other than the intracellular DNA-interacting activity of berberine [209].

### 3.20. Myeloma

In vitro berberine treatment killed multiple myeloma cells and extended the survival of mice bearing multiple myeloma xenografts in vivo. UHRF1 overexpression promoted multiple myeloma cell proliferation and reduced multiple myeloma cells that are more resistant to berberine, although silencing of UHRF1 with siRNA reduced berberine-induced cytotoxicity [210]. Both bortezomib and berberine as single agents showed dose- and time-dependent effects of inhibition of proliferation on multiple myeloma cells. Berberine and bortezomib showed a synergistic effect of proliferation inhibition and early-stage apoptosis proportion in both single agent groups and the combination group. Moreover, berberine plus bortezomib meaningfully enhanced expressions of caspase-3, -8, and -9 [211]. Both berberine and seed-targeting anti-miR-21 oligonucleotide showed a role in the induction of apoptosis, colony inhibition, and G2-phase cell cycle arrest in multiple myeloma cell lines [212]. In human multiple melanoma cell U266, berberine can inhibit the expression of DNMT1 and DNMT3B, which leads to hypomethylation of TP53 by altering the DNA methylation level and the p53-dependent signal pathway. The results suggest that berberine induces apoptosis via hypomethylation of the p53 promoter [213]. Berberine inhibits NF-B nuclear translocation in U266 multiple myeloma cells through Set9-mediated lysine methylation, which results in a drop in miR21 and Bcl-2 levels and triggers ROS production and apoptosis [56] and Berberine inhibited the cell viability of multiple myeloma cells and downregulated the expression of miR-19a/92a [214].

### 3.21. Glioblastoma

Berberine inhibited proliferation and cell viability of human glioblastoma cell lines such as U87 and U251. In the ectopic xenograft model, berberine showed a role in decreasing tumor weight and CD31 mRNA expression in tumor tissue, and in decreasing hemoglobin content in the vehicle group [215]. The treatment of berberine with different concentrations and exposures decreased cell viability of cells of cancer in a concentration-and time-dependent fashion. Next, it was seen that berberine, starting at a concentration of 25 µM exposure, meaningfully increased early apoptosis and suppressed proliferation [216]. Berberine treatment caused enhancement of autophagy and apoptosis in temozolomide-resistant cells. Similarly, after being used in vivo, berberine increased glioblastoma sensitivity to temozolomide via ERK1/2 signaling pathways [217]. In human glioblastoma T98G cells, the relationship between berberine’s antiproliferative properties and the apoptotic pathway connected to its molecular mechanism of action was looked into. Berberine treatment of T98G cell lines decreased cell proliferation and increased cell death in a dose-dependent manner, which was correlated with an increase in G1 arrest. Through the mitochondrial/caspase pathway, berberine caused G1 arrest and apoptosis in human glioblastoma T98G cells. Cell viability of cancer cells was reduced by berberine in a dose-dependent fashion. Berberine increased the level of intracellular Ca^2+^ and the production of reactive oxygen species. Furthermore, berberine also noticeably enhanced apoptosis in cancer cells via activation of caspase-9 and -3 and induction of a higher ratio of Bax/Bcl-2 proteins [218]. By focusing on the AMPK/mTOR/ULK1-pathway, berberine stimulates autophagy in glioblastoma [219].

### 3.22. Melanoma

The proliferation of melanoma cells in a time- and dose-dependent fashion was inhibited by berberine, and cell apoptosis was dose-dependently induced by the same compound. Moreover, low concentrations of berberine caused cell cycle arrest in the G2/M and S phases, whereas high concentrations of berberine showed cell cycle arrest in the G2/M phase [220]. The effect of a combination of cisplatin and berberine-PDT in cisplatin-resistant melanoma cells was investigated. It was reported that cisplatin and berberine-PDT combined treatment triggered the MAPK signaling pathway and inhibition of p38 MAPK [221]. Berberine’s role in melanoma cancer was noted, and it was suggested it may be a valuable adjuvant therapeutic agent in the treatment of melanoma via the PI3K/Akt pathway [187]. K1735-M2 mouse and WM793 human melanoma cells were treated with varying concentrations of berberine, and changes in cell cycle progression, DNA synthesis, cell proliferation, and cell death were measured to test the relationships between berberine uptake, distribution, and cellular effect in melanoma cells. In a melanoma cell line, berberine at various concentrations has different effects on cellular localization patterns and the cell cycle [222]. In melanoma cells, berberine inhibited epithelial mesenchymal transition by regulating PI3K/AKT and RAR/RAR through cross-talk [223]. Berberine showed role in the downregulation the expression level of p-AKT, p-PI3K, and retinoic acid receptor α (RARα) as well as upregulation the expression level of retinoic acid receptor β and γ. These effects of PI3 kinase inhibitor LY294002 treatment mimicked berberine treatment except the expression level of RARγ [224]. 

**Table 2:** Potential of berberine in the prevention and treatment of various types of cancer.

Cancer	In Vitro/In Vivo	Animal/Cell Lines	Dose	Outcome	Refs.
Prostate	In vivo	Xenografts in nude mice	5 mg/kg/day	Berberine treatment administered when tumors reach 100 mm^3^ at a dose of 5 mg/kg/day. Treatment with berberine meaningfully inhibited the growth of the tumor xeongrafts, and this result was confirmed by the final tumor weights.	[113]
Prostate	In vivo	Xenografted into nude mice	5 or 10 mg/kg	The combined treatment of IR and berberine (5 or 10 mg/kg) resulted in tumor growth inhibition of 66.6% and 75.9%, respectively. Together, these data suggest that berberine sensitizes prostate cancer to IR in vivo.	[115]
Prostate	In vitro	PC3 cells	10 μM or 50 μM	Apoptosis rates of the cancer cells treated with berberine were significantly higher and the apoptosis rate increased, survival rates of the PC3 cells in the G2/M phase were high (28.4%) for 50 μM.	[116]
Bladder	In vitro	T24	Different conc. 0, 10, 25, and 50 μg/ml	Berberine at different concentrations inhibited the expression of heparanase at both mRNA and protein levels; the highest decrease in heparanase expression was observed in T24 cells treated with 50 μg/mL of berberine.	[121]
Bladder	In vitro	BIU-87and T24	10 μg/mL and 25 μg/ml	Berberine induces apoptosis and activation of caspase; total apoptotic cells of BIU-87 cells after the berberine treatments were highest for 25 μg/mL (54.40%).	[122]
Kidney	In vitro	ACHN,786-O	Different concentrations (5, 10, 20, 40, 80, 160 and 320 μM)	The cell viability was affected by berberine in a dose-dependent manner (lowest for 320 μM), berberine associated with PDT induces reactive oxygen species generation, autophagy, and apoptosis.	[126]
Liver	In vitro	Huh-7 and HepG2	Different concentration (30–120 μM)	Berberine promotes the p27^Kip1^ and CDKIs expression through regulating the Akt/FoxO3a/Skp2 axis and induces G0/G1 phase cell cycle arrest in a dose-dependent manner (highest for120 μM).	[131]
Liver	In vivo/in vitro	H22, HepG2 and Bel-7404/mice model	Different concentration (0, 50 and 100 *µ*M)	The protein expression levels of cPLA2 and COX-2 was suppressed significantly in a dose-dependent manner at concentrations of 12.5–100 µM. The level of PGE2 was significantly reduced, even when treated with a low dose of BBR (12.5 *µ*M).	[132]
Colon	In vitro	HCT116	Different concentration (0, 1, 10, and 100 µM)	Berberine treatment decreased colon cancer cell viability, as well as induced apoptosis (berberine most suppressed microRNA-21 expression in HCT116 cells at 100 µM).	[136]
Pancreas	In vitro/in vivo	PANC-1, MiaPaCa-2/xenograft in nude mice	Different concentration (0–6 µM)	At a concentration of 3 µM, berberine inhibited DNA synthesis by 82% in MiaPaCa-2 cells and by 76% in PANC-1 cells.	[139]
Pancreas	In vitro	PANC-1 and MIA-PaCa2	Different concentration (0.3–6 mM)	Berberine inhibited cell growth in a dose-dependent manner by inducing cell cycle arrest and apoptosis (highest inhibition by 6 mM barberine).	[140]
Gastric	In vitro	SNU-5	Different concentration (0, 25, 50, 75 and 100 µM)	Berberine seems to show its anticancer activity through inducing ROS production and cell migration prevention. Concentration of 100 µM was potent.	[145]
Gastric	In vivo/in vitro	MKN45, BGC823 and SGC7901/nude mice	Different concentration (15 μM to 90 μM)	Berberine significantly enhanced the activity of cetuximab and erlotinib in vitro and in vivo. Combining berberine in treatment of SGC7901 cells (48 μM) with erlotinib (30 μM) resulted in a 80.5% growth inhibition, compared to 52% growth inhibition for erlotinib alone, a 1.5-fold enhancement.	[147]
Gastric	In vivo/in vitro	SGC7901 and AGS/mice	Different concentration (10–80 μM)	Berberine caused a proliferation inhibition effect, reduced the invasion and migration, and suppressed the gastric cancer tumor growth. Berberine (20 and 30 μM) and MET exhibited the best inhibitory effect on proto-oncogene C-myc in AGS cells.	[148]
	Gastric	MGC 803/nude mice xenografted	Various concentration (0–60 µM)	Berberine time- and dose-dependently inhibited proliferation and suppressed tumorigenesis in nude mice xenografted. Concentration of 60 µM was potent.	[150]
Bile duct	In vitro	KKU-213 and -214	Various concentrations (0–160 µM)	Berberine significantly inhibited growth of Cholangiocarcinoma cell lines. Berberine was reported to suppress proliferation of KKU-213 and -214 at 20 µM than MMNK-1 cells at 120 µM.	[152]
Esophagus	In vitro	KYSE-70 and SKGT4 cells	50 μmol/L	Growth inhibition of cancer cells was noticed by berberine treatment in a dose-dependent and time-dependent fashion. In KYSE-70 cells treated with 50 μmol/L berberine, the number of cells in G_2_/M phase was significantly higher than that in the control group.	[155]
Esophagus	In vitro	KYSE-30	Various concentrations (1, 2, 4, 8, 16, 32, 64, 128 and 256 μM)	The retarded growth, associated with increasing concentrations of berberine, was noticed. In addition, the migration rate of the cells was decreased. The minimum viability was noted for 256 μM.	[156]
Lung	In vitro	A549, PC9, H460, H1299, Beas-2b and 293T cells	Various concentration (0, 40, 80 and 120 μM)	Berberine induces apoptosis through the miR-19a/TF/MAPK signaling pathway, with the most significant increase in cells treated with 80 μM BBR.	[157]
Lung	In vitro	A549 cells	Various concentration (60–200 μM)	Berberine hydrochloride inhibits cell proliferation and promotes the rate of apoptosis; the apoptosis rate was highest in the case of 200 µM.	[164]
Oral	In vitro	HSC-3 cells	Various concentration (5, 10, 25, 50 and 75 µM)	Berberine induced dose- and time-dependent irreversible inhibition of cellular DNA synthesis and cell growth (75 µM showed maximum inhibition).	[167]
Oral	In vitro	KB cells	Various concentration (1, 10, and 100 µM)	Berberine-induced apoptosis might be cyclooxygenase-2-dependent and is related to decreased Mcl-1 expression and Akt phosphorylation; the number of viable cells showed a gradual decrease after 100 µM berberine treatment.	[168]
Cervix	In vitro	HeLa229	Various concentration (3.362, 6.724, 53.791 and 215.164 μM)	Berberine hydrochloride inhibited the proliferation and apoptosis of cancer cells was induced (cell viability was lowest for 215.164 μM berberine hydrochloride treatment), possibly through the downregulation of Bcl-2.	[171]
Cervix	In vitro	HeLa cell l	0.3 mM	Bcl-2/Bax ratio was meaningfully decreased and cytochrome c was released from mitochondrion to cytosol.	[174]
Endometrial	In vitro/in vivo	AN3 CA and HEC-1-A/BALB/cnu/nu athymic mice	50 mg/kg and 100 mg/kg and 25 and 50 μM	Oral administration of berberine at both concentrations of 50 and 100 mg/kg induced significant tumor growth inhibition in tumor models. BBR significantly limited the protein level of COX-2 in a concentration-dependent manner.	[177]
Ovarian	In vitro	OVCAR3 cells and POCCLs	Various concentration (50, 100, 200, 500 μM)	Berberine inhibits cell proliferation and enhances the inhibitory effect of cisplatin in ovarian cancer cells, with the greatest effect at a concentration of 500 μM berberine in both cases.	[181]
Ovarian	In vitro	OVCAR-3 and SKOV-3	Various concentration (1, 10, and 100 µM)	Berberine treatment inhibited the proliferation via cell cycle arrest (% growth inhibition was highest for 100 µM).	[184]
Breast	In vitro	MCF7	Various concentration (1, 10, and 100 µM)	Growth inhibition and induction of cell death was noticed by berberine (% cell death was highest for 100 µM).	[187]
Breast	In vitro/in vivo	4T1 and the human MCF7 cell	Various concentration (50 to 150 μg/Ml)	The weight of tumors decreased with increasing dose of BBR, and the 145 mg/kg BBR dose was most effective in the treatment of breast cancer. BBR induced a dose- and time-dependent decrease in 4T1 cells.	[188]
Breast	In vivo	Rats model	50 mg/kg	Treatment of berberine applied to breast tumor-bearing rats was noticed to be effective against DMBA-induced mammary carcinoma.	[99]
Thyroid	In vitro	8505C and TPC1	10 µM	Berberine treatment of thyroid cancer inhibited the proliferation via apoptosis and/or cell cycle arrest.	[192]
Thyroid	In vitro	C643, OCUT1 and TPC1	Various concentration (0, 10, 20, 40, 80, and 160 μM)	Berberine modulates MAPK, PI3K-AKT signaling pathways in a dose-dependent manner.	[193]
Lymphoma	In vivo/in vitro	DLBCL cell lines/Tumor homograft models	Various concentration (15.30 and 60 μM	Berberine-induced CD47 inhibition increased the phagocytosis of macrophages, thus removing DLBCL cells in vitro and in vivo. A quantity of 30 μM berberine was used to treat U2932, LY1, and LY8 cells; the expression of CD47 was detected and berberine induced downregulation of CD47 in a time-dependent manner.	[196]
Lymphoma	In vivo/in vitro	BC-1, BCBL-1, TY-1/Xenograft mouse model	Various concentration (0, 3, 10, 30 and 100 μM)	Cell proliferation in the primary effusion lymphoma cell lines was inhibited by berberine. In a xenograft mouse model, treatment with berberine inhibited the growth and invasion. The dose of berberine increased from 3 to 100 μM; cell growth inhibition increased in a dose-dependent fashion.	[197]
Bone	In vitro/in vivo	Saos-2 and MG-63/xenograft mouse model	Various concentration (10, 20, 40, 80, 100 and 120 μM)	Berberine significantly inhibits the growth of MG-63 and Saos-2 cells in a time- and dose-dependent fashion. The concentration of berberine at 80 µM inhibited the cell viability to the greatest extent.	[199]
Bone	In vitro	MG-63	Various concentration (2.5, 5, or 10 μM)	Berberine (5 μM) and DDP (2.5 μM) had a synergistic effect at this concentration. Combination treatment of berberine and cisplatin enhanced the inhibition of cell migration and invasion. In addition, combination treatment induced apoptosis and cell cycle arrest in the G0/G1 phase.	[201]
Leukemia	In vivo/in vitro	EU-6 and SKW-3/xenograft mice	Different concentrations (0, 12.5, 25, 50 and 100 μM)	Chemically targeting mTORC1/AKT signaling controls berberine-induced cell autophagy based on in vitro study, and blockade of autophagic process blunts berberine-improved pathological condition in vivo in a dose-dependent manner.	[46]
Leukemia	In vitro/in vivo	KOPN-8, EU-4, NALM-6, EU-6/xenograft mice	Different concentrations (1, 10, 50, 100 and 200 μM)	Berberine reduced acute lymphoblastic leukemia cell viability and induced apoptosis. A quantity of 200 μM BBR induced most significant apoptosis of EU-4 and EU-6 cells. In in vivo studies, berberine significantly improved leukemia conditions in a EU4 xenograft mouse model.	[205]
Myeloma	In vitro	RPMI-8266	Various concentration (75 μM and 120 μM)	Berberine suppresses cancer growth, at least in part, by downregulating miR-21 levels possibly through IL6/)BB, and AMO-miR-21 inhibits cell growth and induces apoptosis; treatment with berberine at 50 μM or higher significantly inhibited cell proliferation STAT3 (most significant for concentrations of 50 μM or higher).	[210]
Myeloma	In vitro	U266 cells	20 μmol/L	Berberine and bortezomib showed synergistic effect of proliferation inhibition.	[211]
Glioblastoma	In vitro	U87MG	Various concentration (10, 25, 100 and 250 μM)	After treatment with several concentrations of berberine, berberine reduced cell viability of cancer cells in a concentration- and time-dependent fashion i.e., highest reduction for 250 μM).	[216]
Glioblastoma	In vitro/in vivo	U87 and U251/Tumor xenograft model	Various concentration (60 and 80 μM)	Berberine has the ability to increase the sensitization of glioblastoma cells to temozolomide treatment in a manner that is dependent upon the ERK1/2-mediated induction of autophagy.	[217]
Melanoma	In vitro	A375	Various concentration (20, 40, 60, and 80 μM)	Berberine suppressed the growth and migration in a dose-dependent manner, i.e., lowest migration for 80 μM berberine treatment.	[220]

## 4. Synergistic Effects of berberine in combination with Other Therapeutic Agents in Cancer Cells

Presently, numerous studies based on in vitro are being undertaken to examine the role of combination therapy using berberine with other natural compounds in the management of cancer through modulating cell signaling pathways (Table 3). An important study found that the combination of berberine with galangin synergistically showed an effect in cell inhibition of growth, cell cycle arrest at the G2/M phase with intracellular reactive oxygen species levels enhancement, and apoptosis in esophageal carcinoma. Moreover, treatment with galangin and berberine alone showed the decreased expressions of β-catenin and Wnt3a. Remarkably, the combination of berberine with galangin can decrease β-catenin and Wnt3a and prompt apoptosis in cancer cells [158]. Synergistic antitumor effects were observed when berberine was employed in combination with other agents to treat hepatomas. The combined use of berberine and evodiamine could significantly enhance the apoptosis of SMMC-7721 cells, which is related to the up-regulation of TNF-α [225].

*d*-limonene or berberine alone can inhibit the growth of human gastric carcinoma cell lines such as MGC803 cells in a dose- and time-dependent fashion. Berberine and *d*-limonene at a 1:4 combination ratio showed a synergistic effect. The two drugs noticeably induced intracellular reactive oxygen species generation, enhanced the expression of caspase-3, decreased the mitochondrial transmembrane potential, and decreased the expression of Bcl-2. The combination of *d*-limonene and berberine presented more noteworthy effects compared with drugs used alone in cancer cells [226]. Curcumin and berberine in combination showed a synergistic inhibitory effect on the growth of both MDA-MB-231 and MCF-7 breast cancer cells compared to the treatment with the compounds alone. Moreover, synergistic anti-breast cancer activities of the combination treatment occurred through the induction of apoptosis and autophagic cell death. The co-treatment-encouraged apoptosis was caspase-dependent and occurred via ERK pathway activation. It was observed that co-treatment of curcumin and berberine strongly decreased phosphorylated Bcl-2 and upregulated phosphorylation of Beclin1 and JNK [42].

Another study reported the synergetic anticancer activity of curcumin and berberine, prompting a cell death percentage of more than 77%, as compared to pure berberine, with <45% and curcumin with <54% on average [227]. S-allyl-cysteine and berberine treatment reduced NF-κβ nuclear translocation via adenylate cyclase-cAMP-protein kinase A axis and finally evaded c-FLIP inhibition. Moreover, S-allyl-cysteine and berberine efficiently decreased Rb-phosphorylation, resulting in an insignificant nuclear E2F presence and leading to the end of cell proliferation [228]. Dose-dependent inhibitory effects on HeLa and A549 cells were noticed in berberine and doxorubicin, which were probably facilitated by inducing apoptosis. Moreover, isobologram illustration and combination index studies have shown that the combination of berberine and doxorubicin causes synergistic effects in cancer cells. These results indicate that berberine sensitizes cells to the anticancer effects of doxorubicin [229]. Berberine can overwhelm doxorubicin resistance in a dose-orchestrated fashion.

By comparison, berberine at a low dose can enhance doxorubicin sensitivity in drug-resistant breast cancer cells. Results confirmed that berberine sensitizes drug-resistant breast cancer to doxorubicin chemotherapy and induces apoptosis via the dose-orchestrated AMPK signaling pathway in vivo and in vitro [191]. In combination with gefitinib in non-small-cell lung cancer, berberine inhibits epithelial–mesenchymal transition [230].

The anticancer effects of berberine in combination with doxorubicin on melanoma cells were investigated based on in vivo and in vitro studies. Combination drugs intensely induced cell death, inhibited cell growth, and caused cell cycle arrest at G2/M. In addition, berberine alone did not show any substantial effect on tumor growth, as seen with doxorubicin, although the combination treatment of both drugs showed a significant and strong decrease in tumor weight and tumor volume as compared to the control [231]. Berberine treatment meaningfully increased cisplatin sensitivity and induced caspase-dependent apoptosis; berberine treatment induced expression of miR-203, and miR-203 overexpression mimicked the cisplatin-sensitizing effect of berberine [232].

Berberine considerably inhibited the OVCAR3 proliferation and primary ovarian cancer cells in a dose- and time-dependent fashion. The combination treatment of berberine and cisplatin showed an induced G0/G1 cell cycle arrest and inhibitory effect on cancer cell growth [181]. Berberine in combination with TRAIL more efficiently induced apoptosis as compared with coptisine, which is structurally related to berberine. In a murine 4T1 breast cancer model, BBR treatment enhanced the efficacy of anti-DR5 antibody therapy against primary tumor growth and lung metastasis [233].

It was observed that ovarian cancer cell line A2780/DDP was incubated with berberine in combination with cisplatin, and showed considerably lower survival than the control group. Berberine increased the cisplatin-induced G0/G1 cell cycle arrest and induced apoptosis in cancer cells [216]. Combination treatment with cisplatin and berberine induced loss of mitochondrial membrane potential and decreased expression of antiapoptotic Bcl-2 and Bcl-x/L. Moreover, cell death induced by the combination treatment was related with increased lipid peroxidation and reactive oxygen species generation. Furthermore, the combined treatment caused apoptosis, which was arbitrated by the caspase cascade activation [234]. Berberine inhibited breast cancer cell growth with a 50% inhibitory concentration, and the IC50 value of cisplatin was 49.541 ± 1.618 µM. However, the IC50 value was noted to be 5.759 ± 0.76 µM for a combination of cisplatin and 26 µM berberine. Berberine sensitized the MCF-7 cells to cisplatin in a time- and dose-dependent fashion. After treatment of berberine and cisplatin, the cleaved capspase-3 and caspase-9 and cellular pro-apoptotic capase-3 were enhanced, and the anti-apoptotic Bcl-2 level was downregulated [39]. The effect of berberine on epirubicin-induced apoptosis, growth inhibition, and cell cycle arrest in bladder cancer cells was examined. It was noticed that berberine increased the inhibitory effect of epirubicin on the viability of bladder cancer cells and endorsed epirubicin-induced cell cycle arrest at G0/G1, in addition to apoptosis [235].

**Table 3:** Combination of berberine with natural compound/anticancer drugs potentially increases the effectiveness of cancer drugs.

Types of Cancer	Berberine Cancer Drugs/Natural Compound	Outcome of the Study	Refs.
Esophagus	Berberine and Galangin	Galangin in combination with berberine showed significant synergistic anti-cancer effects, demonstrating that of both drugs could be hopeful treatment for esophageal carcinoma patients	[225]
Gastric	Berberine and *d*-limonene	Berberine and *d*-limonene combination showed a synergistic effect and enhanced the expression of caspase-3, and decreased the expression of Bcl-2.	[226]
Breast	Berberine and curcumin	Curcumin and berberine in combination showed a synergistic inhibitory effect on the growth of cancer cell and caused induction of apoptosis	[42]
Liver/breast/lung/bone/leukemia	Berberine and curcumin	Synergetic anticancer activity of berberine and curcumin encouraged cell death with greater efficacy as compared to pure curcumin and pure berberine	[227]
Liver	S-allyl-cysteine (SAC) and berberine	S-allyl-cysteine and berberine efficiently decrease Rb-phosphorylation, resulting in insignificant nuclear E2F presence directed to the end of cell proliferation.	[228]
Lung/cervix/liver	Doxorubicin and berberine	Berberine sensitizes cells to the anticancer effects of doxorubicin	[229]
Breast	Doxorubicin and berberine	Berberine overcomes doxorubicin resistance in a dose-orchestrated fashion; berberine also enhances doxorubicin sensitivity in drug-resistant breast cancer cells	[191]
Skin	Doxorubicin and berberine	Combination of doxorubicin and berberine caused a significant decrease in tumor volume and tumor weight	[231]
Gastric	Cisplatin and berberine	Berberine treatment decreases cisplatin resistance of gastric cancer cells through regulating miR-203/Bcl-w apoptotic axis	[232]
Ovarian	Cisplatin and berberine	Combination of both drugs significantly enhanced ovarian cancer cell death via inducing apoptosis and necroptosis	[181]
Ovarian	Cisplatin and berberine	Berberine modulated the sensitivity of cisplatin via the PTEN/miR-93//AKT signaling pathway	[182]
Cervix	Cisplatin and berberine	Combined treatment of cisplatin and berberine induced loss of mitochondrial membrane potential	[235]
Bladder	Berberine and epirubicin	Berberine increased the antiproliferative effects of epirubicin via increasing cell cycle arrest and apoptosis	[236]

## 5. Pharmacokinetics of Berberine

A high-performance liquid chromatographic method for the measurement of berberine in urine, plasma, and bile samples was used. The berberine detection limits in urine, plasma, and bile were 2.3, 18.1, and 90.4 ng/mL, correspondingly. The recoveries of berberine by simple deproteinization of plasma and by solvent extraction of urine were 78.3 and 82.9%, respectively [236]. Berberine was rapidly distributed in the different body organs, such as the liver, kidneys, pancreas, muscle, lungs, heart, brain, and fat, in descending order of its quantity. Moreover, the profile of pharmacokinetics indicated that BBR’s maximum level in the studied tissues was higher than that in plasma four hours after administration. Berberine remained comparatively stable in tissues such as the heart, liver, muscle, pancreas, and brain. Finally, the organ concentration of berberine was higher than its concentration in the blood after administration via the oral route [237]. The main physicochemical properties of berberine and its metabolites were evaluated, including solubility, lipophilicity, pKa, and albumin binding.

An HPLC-ESIMS/MS method was established and confirmed to identify berberine and its chief metabolites in human plasma. Their levels in the plasma of hypercholesterolemic patients and healthy volunteers indicated that berberrubine was found to be the chief metabolite. Amazingly, berberrubine is more lipophilic than berberine, which indicates that this compound tautomerizes to an extremely conjugated, electroneutral quinoid structure [238]. Excretions of berberine and its metabolites in rats were examined after oral administration (200 mg/kg). The total recovered rate of berberine was 22.83% in bile, 22.74% in feces, and 0.0939% in urine. Eighty-three percent of berberine was excreted as thalifendine from bile, whereas berberrubine and thalifendine were the major metabolites, occupying seventy-eight percent of urine excretion. Most berberine and its metabolites were observed in feces, accounting for eighty-four percent of the prototype [239]. The berberine pharmacokinetics and its four metabolites were measured in pseudo-germ-free and conventional rats after p.o. administration with forty mg/kilo gram berberine. The quantities of metabolites were meaningfully decreased in the pseudo-germ-free rats, whereas it was noticed that levels of berberine did not obviously vary among the two groups. The intestinal flora showed no important metabolic activity against berberine and berberine metabolites [240].

## 6. Strategies to Enhance the Berberine Delivery

Natural products are well known for their high potency with minimum side effects. Plant extracts are the most commonly used natural products because of their ease of availability and relatively low production cost [241].

Natural or bioactive compounds have shown a therapeutic role in management of diseases, including cancer, through modulating cell signaling pathways [242,243,244,245,246,247,248]. However, the therapeutic implication of berberine is hindered due to its low absorption, poor bioavailability, low solubility, and quick elimination and metabolism. Nanotechnological-based methods for drug delivery have been used in practice, with nanoformulations comprising berberine appearing to be an appropriate therapeutic approach in numerous cancers [248]. Therefore, several studies have been undertaken to overcome the difficulties of poor absorption, rapid elimination and metabolism, and low solubility. Several reports prove that nanotechnology-based strategies can be used as a delivery strategy to increase the bioavailability of berberine (Table 4).

Anti-solvent precipitation with a syringe pump and evaporative precipitation of nanosuspension were used to overcome the difficulties of the rate of dissolution, solubility, and berberine bioavailability. Results indicate that the nanoparticles enhanced the dissolution rate and solubility due to the change in the crystalline structure to a semi-crystalline form [249]. Lyotropic liquid crystalline nanoparticles were developed to increase the solubility of berberine. A cell viability assay in human breast cancer cells described the formulations prepared with Transcutol^®^ HP and polyethylene glycol-400, and showed a meaningly lower half-maximal inhibitory concentration as compared to pure berberine. Moreover, berberine concentration in Caco-2 cells was greater for lyotropic liquid crystalline nanoparticles prepared with Transcutol^®^ HP [250].

Nanoparticles, such as folate-modified chitosan nanoparticles loaded with berberine hydrochloride (BH/FA-CTS NPs), were prepared to evaluate their role in migration, proliferation, and apoptosis in human nasopharyngeal carcinoma. It was shown that the prepared nanoparticles significantly inhibited the migration and proliferation of cancer cells. Moreover, they induced apoptosis and necrosis of cancer cells and inhibition of tumors was higher [251]. Berberine preloaded into folic acid targeting Janus gold mesoporous silica was prepared to overcome the poor bioavailability of berberine. In vivo and in vitro findings indicated the highly effective antitumor effect, good biosafety, and the effective safety for normal tissue of this nanoplatform [252]. A bioformulation of silver nanoparticles as a carrier for berberine was developed and anticancer activity was evaluated against breast cancer. It was reported that berberine-loaded AgNP complex displays dose-dependent cytotoxicity against breast cancer cells. Moreover, berberine-loaded AgNPs influenced the activation of bax p53 and p53 through downregulating Bcl-2 expression. Histopathological studies and hemolysis assay showed the biocompatibility of the berberine-loaded AgNPs [253].

Berberine was selected as a cancer therapeutic agent and encapsulated on citrate-capped silver nanoparticles. A transmission electron microscopy study displays the cellular invasion of the formulated FA-PEG@BBR-AgNPs, demonstrating the deposit of the nanomaterial at the tumor-specific site. Furthermore, the in vivo antitumor efficacy of FA-PEG@BBR-AgNPs showed a significant limitation of tumor progression, and lesser toxicity of FA-PEG@BBR-AgNPs of brain, liver, lung, kidney, heart, and tissues was verified [254]. Monodisperse silver nanoparticles at a concentration of ten μg/mL arrested cell cycles at the G0/G1 phase in a dose- and time-dependent fashion via disruption of the G0/G1 checkpoint. Silver nanoparticles combined with berberine enhanced the expression of Bcl-2, whereas the ratio of Bax/Bcl-2 decreased in cancer cells. Cancer cells are vulnerable to damage from silver nanoparticle (AgNP)-induced stress, which can be controlled by berberine [255]. Berberine hydrochloride-loaded solid lipid nanoparticles displayed the required drug entrapment efficacy and drug loading, and the release of berberine hydrochloride from SLNs was meaningfully slower than that of free berberine hydrochloride. Notably, in vitro study indicated that Berberine hydrochloride-loaded SLNs more meaningfully inhibited cell proliferation in cancer cells [256].

**Table 4:** Strategies to enhance berberine delivery.

Cancer	Types of Nanoparticles	Outcome	Refs.
Breast	Lyotropic liquid crystalline nanoparticles (LCNs)	LCNs could be a potential carrier for enhancing the solubility and thus improving the anticancer effect.	[250]
Nasopharyngeal	Folate acid modified chitosan nanoparticles berberine hydrochloride (BH/FA-CTS NPs)	BH/FA-CTS NPs indorsed apoptosis and necrosis of CNE-1 cells; BH/FA-CTS NPs showed notably higher tumor inhibition.	[251]
Liver	Folic acid targeting Janus gold mesoporous silica nanocarriers (FA-JGMSNs)	In vitro and in vivo experimental findings exhibited the highly effective antitumor effect, good biosafety, and the effective protection of normal tissue of this nanoplatform.	[252]
Breast	Silver nanoparticles (AgNPs)	Berberine could be straightforwardly loaded to biogenic AgNPs and can assist as a potential anticancer agent for breast cancer.	[253]
Breast	BBR-AgNPs conjugated with polyethylene glycol-functionalized folic acid(FA- PEG): (FA-PEG@BBR-AgNPs)	Formulated nanomaterial can assist as a potential dug-discharging vehicle to battle cancer cells via molecular based targeting approach.	[254]
Tongue	Silver Nanoparticles	Silver particles at low doses therefore decrease the viability and proliferation of oral squamous cell carcinoma cells. SCC-25 cells are vulnerable to injury from AgNPs-induced stress, which can be controlled by the natural alkaloid berberine.	[255]
Breast/liver/lung	Solid lipid nanoparticle	Solid lipid nanoparticles formulation may serve as a new, simple, and effective system for the delivery of berberine hydrochloride.	[250]
	Solid lipid nanoparticles		
Lung/skin	Lipid based nanoparticles	Moderately cytotoxic dose of BBM-NPs was able to significantly suppress the incidence of B16F10 cells lung metastasis in vivo. Suppression of primary B16F10 melanoma tumor growth in C57BL/6 mice model treated with BBM-NPs compared to that of native BBM.	[257]

## 7. Conclusions

Berberine is a bioactive compound that is extracted from numerous plants. The molecular understandings of berberine’s pro-death effects on cancer cells are still unknown, despite numerous investigations into its targets and modes of action. The experimental findings based on in vitro and in vivo studies indicate that berberine shows health-promoting effects through its antioxidant, anti-inflammatory, anticancer, and immunomodulatory properties. Berberine reveals anticancer potential by directing cell signaling pathways, such as angiogenesis, cell cycle, apoptosis, autophagy, transcription factors, and other cell signaling molecules. In vitro-based studies indicate the efficacy and synergistic effect of berberine in disease management in combination with cancer agents or natural products. Recent studies have shown that berberine can be used in combination with chemotherapy agents, even though the precise mechanism of action of berberine as an anticancer drug has not yet been fully explained and more research is required. It has also been emphasized that berberine has the capacity to boost chemosensitivity and lessen the negative effects of chemosensitizers. Cancer therapy may benefit from the use of berberine and its derivatives, which may increase clinical efficacy and safety. The native berberine has shown a therapeutic role in cancer management but the therapeutic implication of berberine is hindered due to its low absorption and poor bioavailability. Therefore, several studies have been undertaken to overcome the difficulties of low absorption and poor bioavailability through nanotechnology-based strategies.

## Data Availability

The data used to support the findings of this study are included within the article.
